# Impact of fluorescence angiography on anastomotic leak and complication rate in colorectal surgery: A systematic review and meta‐analysis of randomized controlled trials

**DOI:** 10.1111/codi.70236

**Published:** 2025-10-01

**Authors:** Alexandre Balaphas, Marwan Julien Sleiman, Guillaume Meurette, Emilie Liot, Christian Toso, Frédéric Ris, Jeremy Meyer

**Affiliations:** ^1^ Division of Digestive Surgery University Hospitals of Geneva Genève Switzerland; ^2^ Department of Surgery University of Geneva Geneva Switzerland

**Keywords:** anastomotic leak, angiography, bowel division, colectomy, colorectal surgery, ischemia, prevention

## Abstract

**Objective:**

Anastomotic leak occurs in 8.1% of right colectomies and up to 17.1% of low anterior resections. Fluorescence angiography has gained acceptance in recent years as a method for assessing anastomosis vascularization, a key element implicated in anastomotic leak. Our objective was to perform a systematic review and meta‐analysis of randomized controlled trials on the effect of fluorescence angiography on anastomotic leak and postoperative morbidity.

**Methods:**

A systematic review was performed on Medline, Embase and CENTRAL according to the PRISMA statement until 16 March 2025. Randomized controlled trials in English that compared fluorescence angiography with standard care were considered eligible. Articles were screened, bias was detected, data were extracted, pooled and analysed.

**Results:**

Among 477 identified studies, 401 were retained for screening but only eight were included in the quantitative analysis (3999 patients). Fluorescence angiography was significantly protective against anastomotic leak, with an odds ratio of 0.64 (95% CI: 0.39–0.98, *I* ^2^: 0%, *p* < 0.0001) and a reduction in risk of 4 percentage points (95% CI: −0.05 to 0.02, *I* ^2^: 0%, *p* < 0.0001). When analysis was restricted to colorectal anastomosis, the effect of fluorescence angiography on anastomotic leak was maintained (OR 0.59, 95% CI 0.44–0.79, *I* ^2^: 0%, *p* < 0.0005). However, fluorescence angiography did not reduce postoperative morbidity compared with the control group.

**Conclusion:**

High‐quality evidence shows that fluorescence angiography reduces the rate of anastomotic leak in colorectal surgery with a decrease in the incidence of 4 percentage points.

## INTRODUCTION

Anastomotic leak remains one of the most serious complications following colorectal resection. The incidence of this complication varies depending on the location of the anastomosis: the lower the anastomosis, the higher the risk [1]. Until recently, the reported risk of anastomotic leak after hemicolectomy was 8.1%, whereas for low rectum surgery, an incidence of 17.1% was reported in the GRECCAR5 trial [2, 3]. Anastomotic leak significantly increases postoperative morbidity, healthcare costs and mortality [4]. Moreover, anastomotic leak has a direct impact on oncological outcomes, as patients with colorectal cancer who experience an anastomotic leak are at a higher risk of recurrence [5]. This is largely due to delays in the initiation of adjuvant chemotherapy, which often must be postponed in the event of a leak.

Multiple factors contribute to the risk of anastomotic leak, including patient‐related factors, the composition of the intestinal microbiota and surgical technique [4]. Over the past decades, considerable efforts have focused on optimizing intraoperative assessment of bowel perfusion—a critical factor in preventing anastomotic failure [6]. Indeed, adequate perfusion of each bowel edge is crucial for the healing process of the anastomosis [7]. Traditionally, various methods have been employed to evaluate tissue perfusion and prevent ischaemia, such as visual inspection, Doppler ultrasonography and measurement of tissue oxygenation. However, these techniques are often impractical during minimally invasive procedures [7]. Fluorescence angiography has emerged as a valuable tool in this context. This technique involves the intravenous injection of a fluorochrome, such as indocyanine green, which enables real‐time visualization of blood vessels under near‐infrared light. Indocyanine green is particularly well suited for this purpose due to its safety profile, rapid elimination and superior performance compared to other dyes [8]. As a result, fluorescence angiography has become a standard method for assessing anastomotic perfusion during laparoscopic surgery.

Over time, intraoperative FA has been increasingly utilized to evaluate anastomotic perfusion, and its effectiveness has been investigated in several studies [9]. In a previous systematic review and meta‐analysis of three randomized controlled trials (RCTs), we found that the use of FA was associated with a modest reduction in the rate of anastomotic leaks [9]. However, our earlier analysis was limited by the small number of included studies, which focused exclusively on colorectal anastomoses and considered only a single primary outcome. To address these limitations, we undertook a new systematic review and meta‐analysis, incorporating the most recent data across all types of anastomoses. This study aimed to provide a comprehensive assessment of the impact of FA on both anastomotic leak rates and overall complication rates, incorporating the most recent RCT data and representing the largest pooled analysis to date.

## MATERIALS AND METHODS

This systematic review was conducted in accordance with the 2020 PRISMA guidelines [10] and registered in Prospero (1055070). The research was run on MEDLINE, Embase and CENTRAL to 16 March 2025 for English‐language written RCTs evaluating the effects of fluorescence angiography compared with standard assessment, as a control, on the occurrence of anastomotic leak (Table 1). Non‐randomized studies, conference abstracts, letters and secondary analyses were excluded. All methods for performing fluorescence angiography were accepted. The screening and inclusion of eligible articles were performed by two independent reviewers (AB and JM). A consensus was reached with a third reviewer when necessary (MJS). Data were extracted from selected articles, and details on anastomotic leak definition and detection were recorded. For the purposes of this meta‐analysis, any anastomotic leak, regardless of its classification or management, was considered a positive event. Postoperative morbidity (30 or 90 days) was defined as a Clavien–Dindo score [11] ≥3a. The postoperative morbidity variable was recoded according to this definition. Pooled odds ratio (OR) and pooled risk difference (RD) values were obtained via models with random effects. Heterogeneity was assessed via the Q test and quantified using the *I* ^2^ value. A sensitivity analysis was performed, including only studies with colorectal anastomosis. The risk of bias was assessed using the revised Cochrane risk‐of‐bias tool for randomized trials (RoB2) [12]. The online tool Rayyan (Cambridge, MA, USA) was used for article selection, Microsoft Excel 2021 (Redmond, WA, USA) was used for data extraction, and RevMan was used for data analysis (Review Manager Web tool, V. 9.3.0 2 June 2025, The Cochrane Collaboration).

**TABLE 1:** Strategy search.

Database	Search build	Occurrences
MEDLINE	((Near‐infrared) OR (NIR) OR (indocyanine green) OR (ICG) OR (angiography) OR (fluorescence angiography)) AND ((Colorectal) OR (colectomy)) AND (“Anastomotic Leak”[MeSH Terms] OR (anastomosis leakage) OR (leak) OR (insufficiency) OR (reoperation))	308
EMBASE	(‘fluorescence angiography’/exp. OR ‘fluorescence angiography’) AND (‘anastomotic leak’/exp. OR ‘anastomotic leak’) AND ‘controlled study’/de AND ‘human’/de	121
COCHRANE CENTRAL	(*fluorescence angiography) AND (anastom*)	46
Other sources	–	2

## RESULTS

### Inclusion process

**FIGURE 1:**
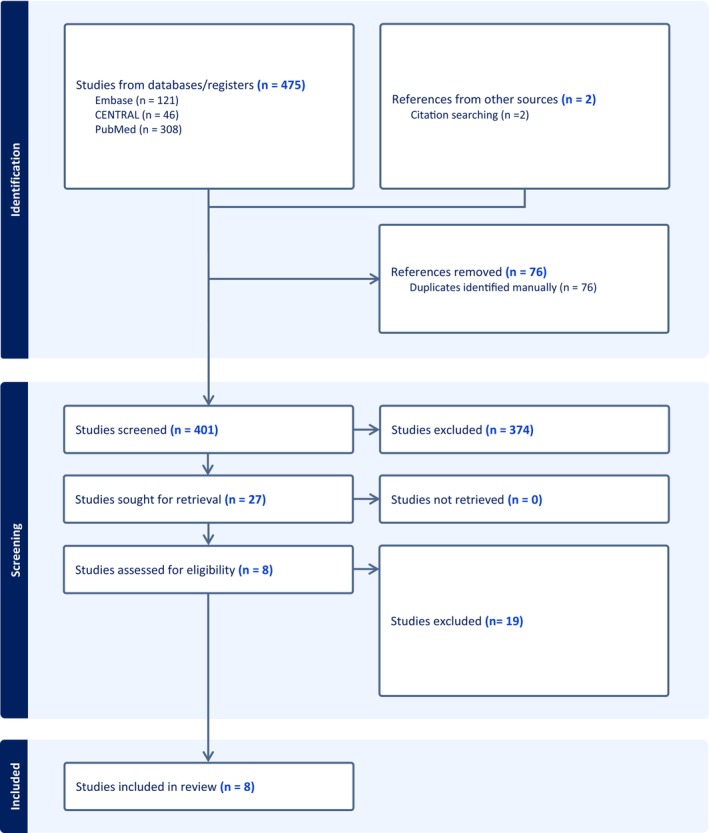
Flow chart according to the PRISMA 2020 statement.

Through research on MEDLINE, CENTRAL and EMBASE, 475 articles were identified, and two additional articles were identified via reference screening. After duplicate removal, 401 studies were retained for screening on the basis of titles and abstracts, and eight were finally retained (Figure 1) [13, 14, 15, 16, 17, 18, 19, 20]. The included articles used the near‐infrared technology indocyanine green. All the articles reported results of intention‐to‐treat analysis except one [20]. Two articles did not restrict their inclusion criteria to colorectal anastomoses and included patients with different types of anastomoses, such as colo‐colic or ileo‐colic anastomoses [17, 20].

### Characteristics of the included studies

Included studies were published over the last 5 years (2020–2025) with a sample size ranging from 85 to 1136 patients (Table 2). Studies were from Russia [13], Italy [15], USA [14], Poland [16], Egypt [18], Japan [17], Netherlands [19] and Finland [20]. Half of the retained RCTs included both malignant and benign cases [13, 15, 19, 20], whereas the other half were restricted to malignant disease [14, 16, 17, 18]. All eight studies used anastomotic leak as the primary outcome and almost all studies used the definition of the International Study Group of Rectal Cancer (Table S1) [21]. However, the time set was different among the trials. Six out of eight studies used a 30‐day definition [13, 15, 16, 17, 18, 20], one trial used a 60‐day definition [14], and the other used a 90‐day definition [19]. The results of the quality assessment of the articles according to the revised Cochrane Collaboration's risk of bias tool (RoB2) are reported in Table 3.

**TABLE 2:** Details of the reviewed studies.

Author(s)	Year	Country	Acronym	Period	*n*	Population	Intervention	Control	Primary outcome
Alekseev et al.	2020	Russia	FLAG	11.2017‐08.2019	377	Elective stapled colorectal anastomosis located below 15 cm from the anal verge for benign or malignant disease	ICG fluorescence angiography	Macroscopic appearance of the bowel	Anastomotic leak < 30 days
De Nardi et al.	2020	Italy	NA	01.2016‐11.2017	240	Elective stapled colorectal anastomosis located 2–15 cm from the anal verge for benign or malignant disease	ICG fluorescence angiography	Macroscopic appearance of the bowel + perfusion of marginal blood vessels	Anastomotic leak < 30 days
Jafari et al.	2021	USA	PILLAR III	03.2015–02.2017	347	Elective stapled colorectal anastomosis located up to 10 cm from the anal verge for malignant disease	ICG fluorescence angiography	No fluorescence angiography	Anastomotic leak < 8 weeks
Gach et al.	2023	Poland	NA	12.2020–08.2021	85	Elective stapled colorectal anastomosis located up to 12 cm from the anal verge for malignant disease	ICG fluorescence angiography	Macroscopic appearance of the bowel	Anastomotic leak < 30 days
Watanabe et al.	2023	Japan	EssentiAL trial	12.2018–02.2021	850	Elective colorectal anastomosis located up to 12 cm from the anal verge for malignant disease	ICG fluorescence angiography	Macroscopic appearance of the bowel + perfusion of marginal blood vessels	Anastomotic leak < 30 days
Eltaweel and Mohamadain	2024	Egypt	NA	01.2022–10.2022	101	Elective manual colorectal anastomosis located 2–10 cm from the anal verge for malignant disease	ICG fluorescence angiography	Macroscopic appearance of the bowel + perfusion of marginal blood vessels	Anastomotic leak < 30 days
Faber et al.	2024	Netherlands	AVOID	07.2020–02.2021	982	Elective colorectal, colo‐colic or ileo‐colic anastomosis for malignant or benign disease	ICG fluorescence angiography	Macroscopic appearance of the bowel	Anastomotic leak < 90 days
Rinne	2025	Finland	ICG‐COLORAL	09.2018–12.2023	1136	Elective colorectal above peritoneal reflexion line, colo‐colic or ileo‐colic anastomosis for malignant or benign disease	ICG fluorescence angiography	Macroscopic appearance of the bowel + perfusion of marginal blood vessels	Anastomotic leak < 30 days

Abbreviations: ICG, indocyanine‐green; NA, not applicable.

**TABLE 3:** risk of bias according to rob2.

Analyzis	Study	Outcome	D1	D2	D3	D4	D5	Overall
ITT	Alekseev et al.	Anostomic leak	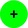	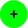	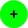	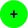	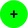	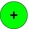
ITT	De Nardi et al.	Anostomic leak	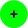	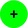	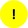	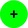	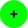	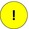
ITT	Jafari et al.	Anostomic leak	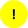	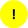	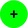	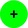	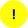	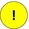
ITT	Gach et al.	Anostomic leak	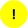	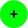	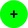	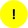	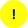	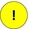
ITT	Eltaweel and Mohamadain	Anostomic leak	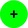	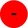	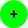	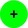	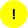	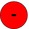
ITT	Watanabe et al.	Anostomic leak	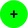	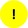	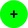	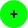	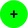	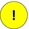
ITT	Faber et al.	Anostomic leak	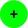	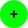	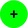	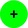	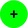	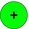
PP	Rinne et al.	Anostomic leak	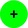	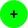	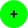	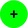	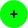	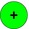
ITT	Alekseev et al.	Morbidity	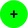	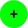	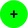	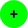	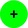	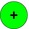
ITT	De Nardi et al.	Morbidity	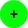	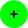	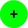	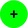	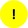	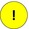
ITT	Gach et al.	Morbidity	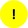	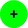	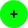	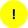	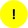	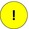
ITT	Eltaweel& Mohamadain	Morbidity	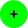	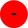	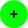	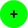	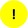	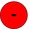
ITT	Watanabe et al.	Morbidity	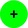	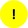	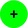	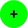	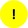	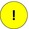
PP	Rinne et al.	Morbidity	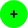	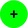	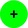	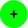	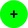	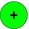

*Note*: Green dot: low risk; Red dot: high risk; Yellow dot: some concerns.

Abbreviations: D1, randomisation process; D2, deviations from the intended interventions; D3, missing outcome data; D4, measurement of the outcome; D5, selection of the reported result; ITT, intention to threat; PP, per protocol.

### Effect of fluorescence angiography on anastomotic leak after colorectal surgery

**FIGURE 2:**
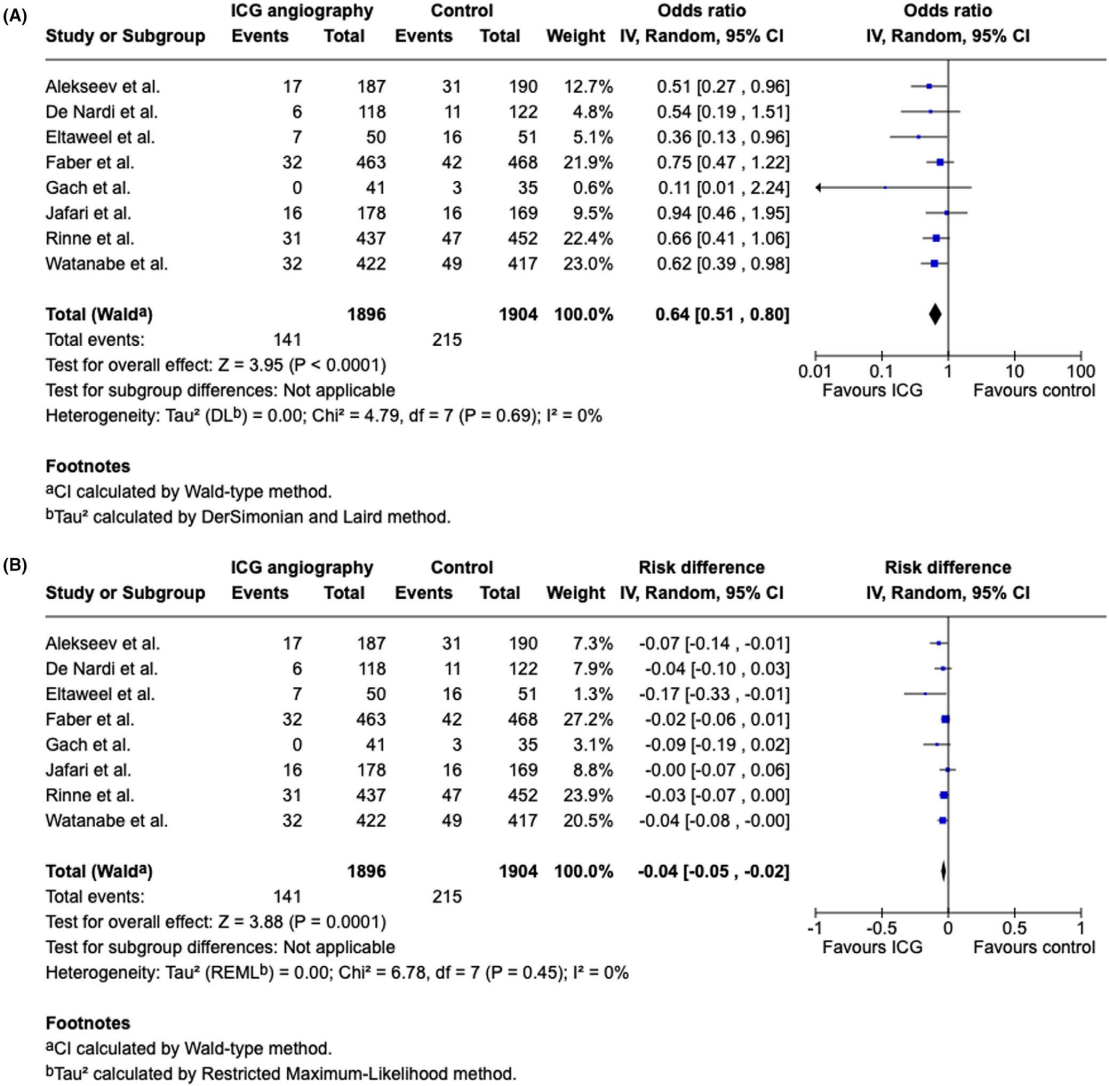
Meta‐analysis of fluorescence angiography versus the control for anastomotic leak expressed as the odds ratio. Forest plot comparing fluorescence angiography versus the control for anastomotic leak. Each horizontal bar summarizes a study. The bars represent 95% confidence intervals. The grey squares indicate each of the studies' weights in the meta‐analysis. The diamond in the lower part of the graph depicts the pooled estimate along with 95% confidence intervals. (A) Results are represented as odds ratios. (B) Results represented as risk differences. The odds ratio (OR) was obtained via models with random effects (Mantel–Haenszel). Heterogeneity was assessed via the *Q* test and quantified via the *I* ^2^ value.

All eight eligible articles, which represented a total of 3999 patients, were included in the primary outcome meta‐analysis. Fluorescence angiography was statistically significantly protective against anastomotic leak with an OR of 0.64 (95% CI: 0.39–0.98, *I* ^2^: 0%, *p* < 0.0001; Figure 2A). Additionally, when fluorescence angiography was performed, the incidence of anastomotic leak was statistically significantly decreased by 4 percentage points (95% CI: −0.05 to 0.02, *I* ^2^: 0%, *p* < 0.0001; Figure 2B).

### Effect of fluorescence angiography on postoperative morbidity after colorectal surgery

**FIGURE 3:**
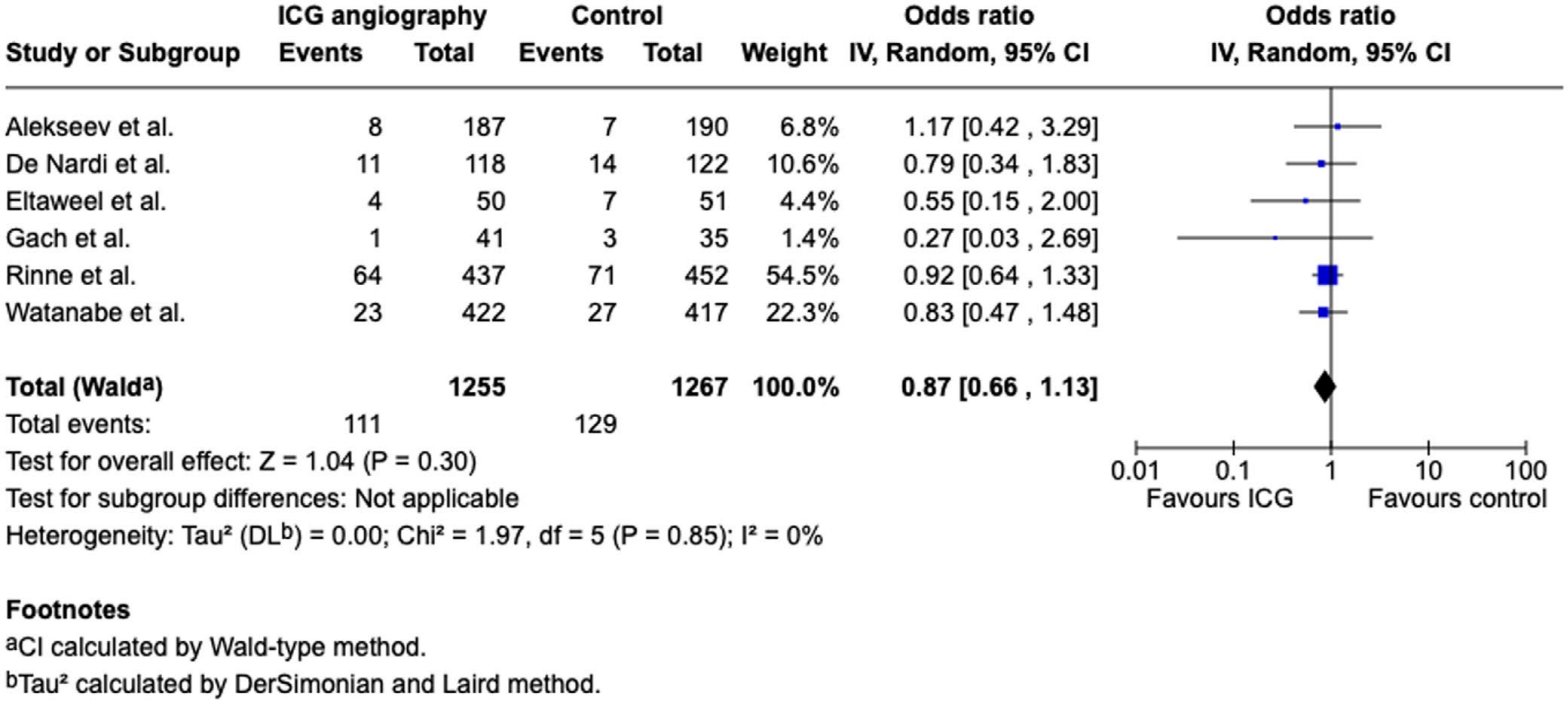
Meta‐analysis of fluorescence angiography versus the control for postoperative morbidity expressed as difference risk. Forest plot comparing fluorescence angiography versus the control for morbidity (Clavien–Dindo score ≥ 3a). Each horizontal bar summarizes a study. The bars represent 95% confidence intervals. The grey squares indicate each of the studies' weights in the meta‐analysis. The diamond in the lower part of the graph depicts the pooled estimate along with 95% confidence intervals. The odds ratio (OR) was obtained via models with random effects (Mantel–Haenszel). Heterogeneity was assessed via the *Q* test and quantified via the *I* ^2^ value.

The secondary outcome meta‐analysis was restricted to six out of eight articles, as comprehensive data on complications were not available [13, 14, 15, 16, 17, 18]. Notably, two studies extended the surveillance period for complications to 60 (Jafari et al.) and 90 days (Faber et al.) [14, 19]. Compared with standard bowel evaluation, fluorescence angiography did not appear to significantly reduce postoperative morbidity (Figure 3; *n* = 1255, OR 0.87, 95% CI: 0.66–1.13, *I* ^2^: 0%, *p* < 0.30).

### Sensitivity analysis

A sensitivity analysis of primary and secondary outcomes was performed by including only studies where a colo‐rectal anastomosis was performed, eliminating the studies of Faber et al. and Rinne et al. that considered all types of anastomosis (Figure S1A,B) [19, 20]. Indeed, these studies only distinguish between right‐sided and left‐sided anastomosis. This reduced the number of included patients to 1980. With this parameter, both the OR and RD of anastomotic leak were similar to those of the initial analysis (OR 0.59, 95% CI 0.44–0.79, *I* ^2^: 0%, *p* < 0.0005; RD −0.05, 95% CI −0.08 to −0.00, *I* ^2^: 0%, *p* < 0.0006). Moreover, the effect of fluorescence angiography on postoperative morbidity was still non‐significant (818 patients, OR 0.80, 95% CI 0.54–1.20, *I* ^2^: 0%, *p* < 0.29; Figure S2).

## DISCUSSION

In this work, we have shown that the application of fluorescence angiography is linked to a reduction in anastomotic leak rates by approximately 4 percentage points, without a significant impact on overall morbidity. These findings are consistent with previous meta‐analyses, although those reviews were based on different data sets [9, 22]. For example, Elmajdub et al. [22] recently reported an odds ratio of 0.55 (95% CI: 0.35–0.87, *p* = 0.012) regarding anastomotic leak when fluorescence angiography was performed, with five studies included, two in Chinese. The authors did not find a statistically significant effect of fluorescence angiography on specific complications. However, they did not include the recently published ICG‐COLORAL trial (Rinne et al.), which recruited 1136 patients [20].

The risk of bias across the included studies in our own analysis was a point of concern, but we chose not to exclude any studies on this basis. This approach, while potentially allowing a few studies to disproportionately influence the results, was deemed necessary given the limited number of RCTs available on this topic. Moreover, although numerous parameters may influence the occurrence of anastomotic leak, low heterogeneity was observed in our analysis, with an *I* ^2^ of 0%. Also, funnel plot representation demonstrated a symmetrical and pyramid‐shaped distribution of the included studies (Figure S3).

Among the earliest RCTs published, De Nardi et al. [15] observed a reduction in anastomotic leak (9% vs. 5%) in a cohort of 240 patients, but this difference was not statistically significant. Similarly, Gach et al. [16], with 86 patients, reported a decrease in anastomotic leak (0% vs. 8.6%) with the use of fluorescence angiography, though statistical significance was not achieved. Additionally, Jafari et al. [14] did not find any statistically significant difference between the fluorescence angiography group and the control group in 347 patients. However, the study was terminated before the calculated minimal number of 450 participants was reached. In contrast, Alekseev et al. (*n* = 377), Eltaweel and Mohamadai (*n* = 101) and Watanabe et al. (*n* = 850) all reported statistically significant reductions in anastomotic leak [13, 17, 18]. However, these results contradict those of the larger and more recent AVOID and ICG‐COLORAL trials, which included 982 and 1136 participants, respectively.

Although the level of statistical significance varied among these studies, each demonstrated some preventive effect of fluorescence angiography on anastomotic leak. This variability may be explained by differences in baseline leak rates, which are known to vary widely across institutions and regions. Anastomotic leak is influenced by local protocols, and where enhanced recovery practices are well established, leak rates tend to be lower. For instance, our own unpublished data indicate a leak rate of 3.08% for low rectal anastomoses when fluorescence angiography is included in our standard protocol [23, 24].

The studies that reported the most pronounced benefits of fluorescence angiography also documented baseline leak rates above 10%. Watanabe et al. [17] used a control arm leak rate of 13% in their sample size calculation, with the actual rate in the study being 11.8%. Alekseev et al. and Eltaweel and Mohamadai reported control arm leak rates of 16.3% and 16%, respectively [13, 18]. Conversely, studies that found no significant effect of fluorescence angiography reported control arm leak rates ranging from 8.3% to 9.6% [14, 15, 19, 20]. This suggests that anastomotic leak is generally a rare event, and in centres with robust protocols, it may be challenging to further reduce its occurrence to a point where factors other than ischaemia become predominant. In other words, the benefit of fluorescence angiography may be most evident in settings where other risk factors for anastomotic leak are not as well controlled. Other factors, such as the method of leak detection and the routine use of protective loop ileostomy, may also influence reported leak rates.

Regarding morbidity, our secondary analysis found a slight decrease when fluorescence angiography was used, but this difference was not statistically significant. This may be due to the fact that the included studies were not specifically designed to detect differences in morbidity and were likely underpowered for this outcome. Moreover, while anastomotic leak remains a serious complication of colorectal surgery, current management strategies—such as antibiotics, a watch‐and‐wait approach, and preventive ileostomy—can mitigate its severity and reduce the need for invasive interventions.

Despite the effect being less dramatic than some might have hoped, we believe that fluorescence angiography does have a role in preventing anastomotic leak. Future large‐scale trials should focus on high‐risk colorectal procedures and pragmatic outcomes, such as length of hospital stay, to better demonstrate its impact. Moreover, it may be of interest to choose as primary outcome and/or to report dynamic AF parameters such as the time necessary to obtain a signal.

The topic remains of significant interest, and results from ongoing and upcoming trials (NCT06793280, NCT06708819, NCT06845306 [ICG‐NCF] and NCT05168839 [FLUOCOL‐1] [25]) are eagerly awaited. However, as fluorescence angiography becomes standard practice in many centres, the feasibility of conducting new RCTs may be limited. The procedure is safe, adds only a few minutes to the operation, and is minimally costly, particularly as most modern laparoscopic systems are equipped for near‐infrared detection [26].

## CONCLUSION

The use of fluorescence angiography significantly reduces the rate of anastomotic leak during colorectal surgery but does not significantly impact postoperative morbidity. While the effect size is limited by the rarity of anastomotic leakage, new RCTs are ongoing that could reinforce this observation for anastomotic leak and perhaps other outcomes, such as postoperative morbidity.

## AUTHOR CONTRIBUTIONS

**Alexandre Balaphas:** Conceptualization; methodology; data curation; investigation; writing – original draft; writing – review and editing; project administration. **Marwan Julien Sleiman:** Data curation; writing – review and editing; validation. **Guillaume Meurette:** Writing – review and editing; supervision. **Emilie Liot:** Supervision; writing – review and editing. **Christian Toso:** Supervision; writing – review and editing. **Frédéric Ris:** Conceptualization; writing – review and editing; supervision; project administration. **Jeremy Meyer:** Conceptualization; methodology; project administration; writing – review and editing; visualization; validation; supervision.

## FUNDING INFORMATION

There was no funding for this research.

## CONFLICT OF INTEREST STATEMENT

The authors disclose no conflicts of interest.

## ETHICS STATEMENT

As it was a meta‐analysis of already published studies, the project was not presented to the IRB.

## Supporting information


Figure S1.

Figure S2.

Figure S3.

Table S1.

Data S1.

## Data Availability

The data that support the findings of this study are available from the corresponding author upon reasonable request.
