# Supplementary figures and images for: Impact of fluorescence angiography on anastomotic leak and complication rate in colorectal surgery: A systematic review and meta‐analysis of randomized controlled trials
Source: Colorectal Dis. 2025 Oct 1;27(10):e70236. doi: 10.1111/codi.70236 (PMC12485866; doi:10.1111/codi.70236)

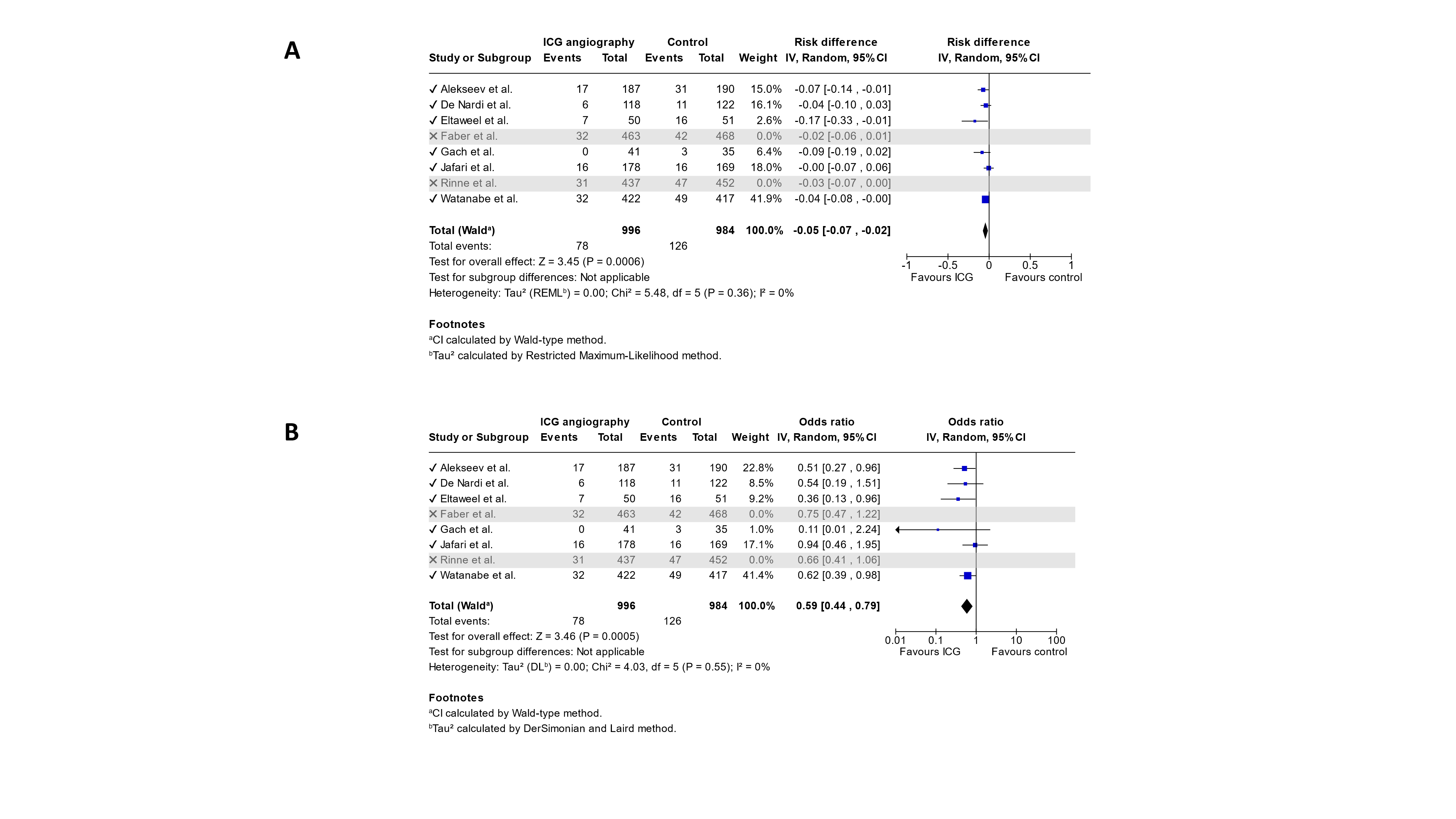

Supplement: Supplementary file 1 — Figure S1. [file CODI-27-0-s004.tif]

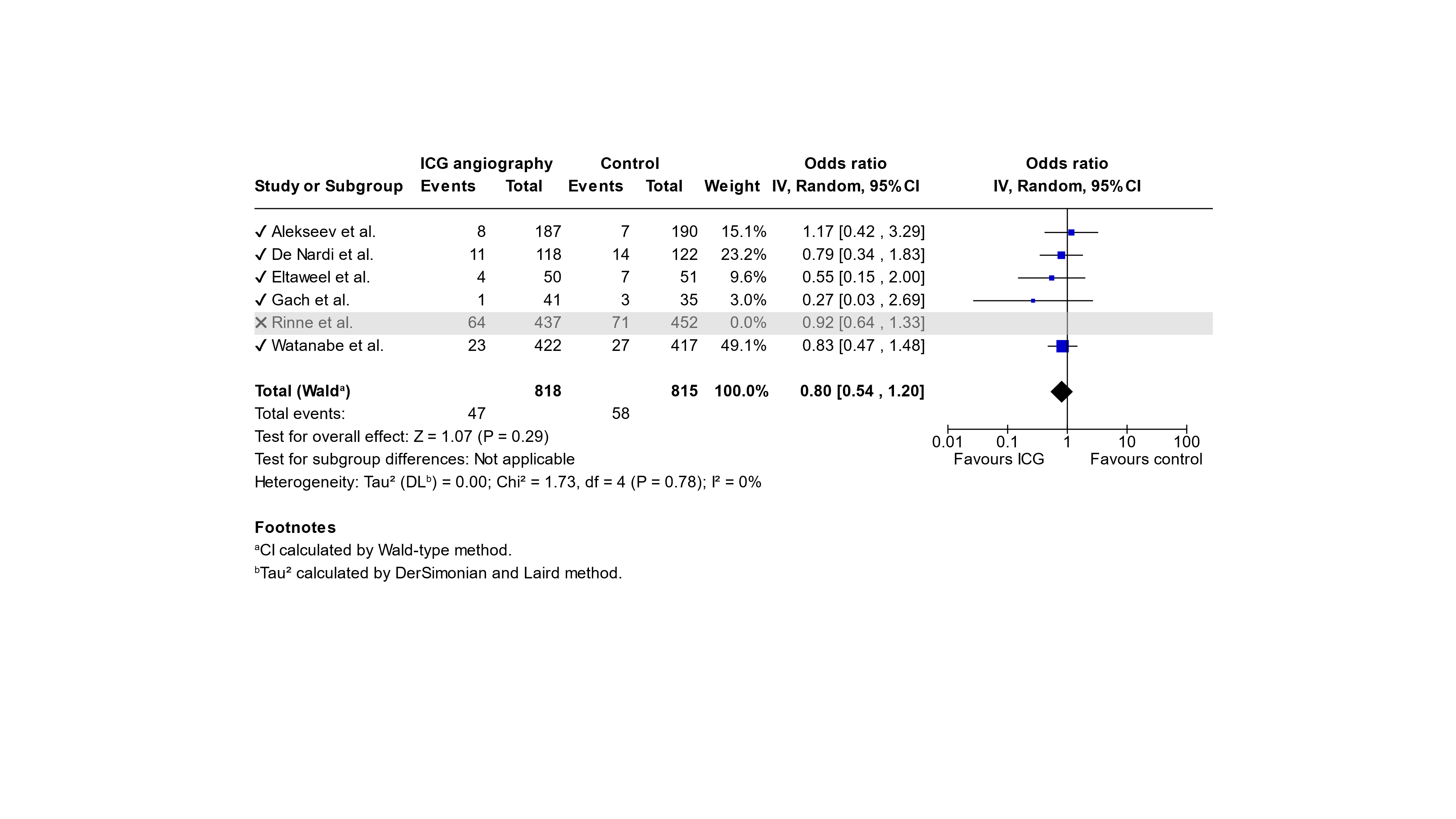

Supplement: Supplementary file 2 — Figure S2. [file CODI-27-0-s001.tif]

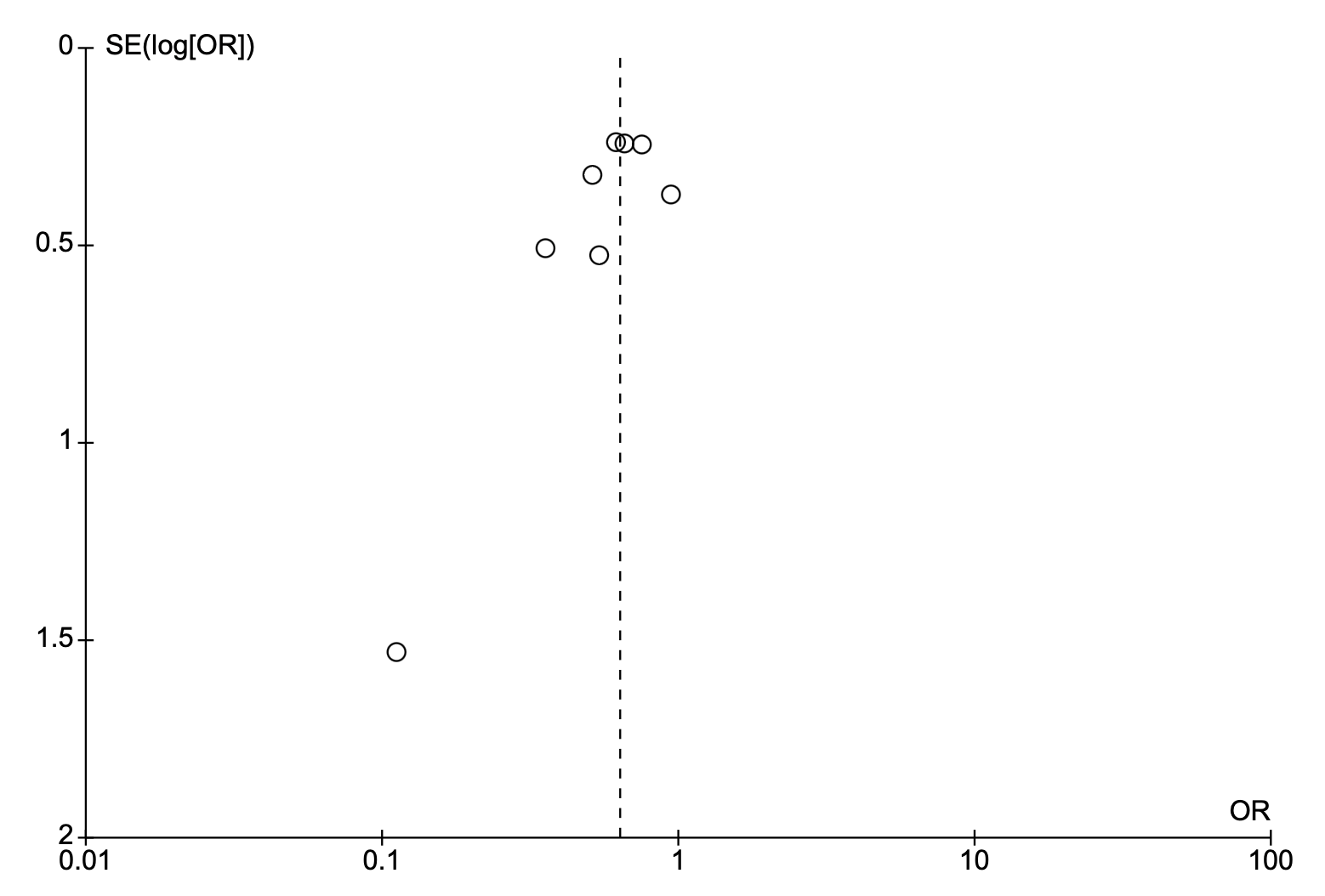

Supplement: Supplementary file 3 — Figure S3. [file CODI-27-0-s005.tif]
