# Supplementary material for: Impact of fluorescence angiography on anastomotic leak and complication rate in colorectal surgery: A systematic review and meta‐analysis of randomized controlled trials
Source: Colorectal Dis. 2025 Oct 1;27(10):e70236. doi: 10.1111/codi.70236 (PMC12485866; doi:10.1111/codi.70236)
Supplement: Supplementary file 4 — Table S1. [file CODI-27-0-s002.docx]

| Authors | Year | Definition of anastomotic leak | Detection of anastomotic leak | Available classification of anastomotic leak | Systematic radiological assessement |
| --- | --- | --- | --- | --- | --- |
| Alekseev et al. | 2020 | International  Study Group of Rectal Cancer | Clinical, endoscopic and/or radiological evaluation | Yes | Yes |
| De Nardi et al. | 2020 | International  Study Group of Rectal Cancer | Clinical, endoscopic and/or biological suspicion confirmed by a CT scan | Yes | No |
| Jafari et al. | 2021 | Any evidence  of endoluminal contents (air, fluid, GI contents, or contrast  material) | Clinical, biological and/or radiological evaluation | No | Yes |
| Gach et al. | 2023 | International  Study Group of Rectal Cancer | Clinical and/or endoscopic evaluation | No | No |
| Watanabe et al. | 2023 | International  Study Group of Rectal Cancer | Suspicion confirmed by radiological evaluation | Yes | Only for diverting stoma |
| Eltaweel& Mohamadain | 2024 | International  Study Group of Rectal Cancer | Clinical or biological suspicion confirmed by endoscopic, radiologic or surgical evaluation | No | No |
| Faber et al. | 2024 | International  Study Group of Rectal Cancer | Not detailed | No | No |
| Rinne | 2025 | Not specified | Suspicion confirmed by radiological evaluation | No | No |

Table S1. Details of the reviewed studies regarding definition and detection of anastomotic leak.
