# Supplementary material for: Impact of fluorescence angiography on anastomotic leak and complication rate in colorectal surgery: A systematic review and meta‐analysis of randomized controlled trials
Source: Colorectal Dis. 2025 Oct 1;27(10):e70236. doi: 10.1111/codi.70236 (PMC12485866; doi:10.1111/codi.70236)
Supplement: Supplementary file 5 — Data S1. [file CODI-27-0-s003.docx]

**Figure S1. Sensitivity analysis of the meta-analysis of fluorescence angiography versus the control for anastomotic leakage**. Forest plot comparing fluorescence angiography versus the control for anastomotic leakage after the exclusion of studies considering anastomosis with the colon and small bowel and colon. Each horizontal bar summarizes a study. The bars represent 95% confidence intervals. The grey squares indicate each of the studies' weights in the meta-analysis. The diamond in the lower part of the graph depicts the pooled estimate along with 95% confidence intervals. (A) Results are represented as odds ratios. (B) Results are represented as risk differences. The odds ratio (OR) was obtained via models with random effects (Mantel–Haenszel). Heterogeneity was assessed via the Q test and quantified via the I^2^ value.

**Figure S2. Sensitivity analysis of the meta-analysis of fluorescence angiography versus the control for morbidity**.
Forest plot comparing fluorescence angiography versus the control for morbidity after the exclusion of studies considering anastomosis with the colon and small bowel and colon. Each horizontal bar summarizes a study. The bars represent 95% confidence intervals. The grey squares indicate each of the studies' weights in the meta-analysis. The diamond in the lower part of the graph depicts the pooled estimate along with 95% confidence intervals. (A) Results are represented as odds ratios. (B) Results represented as risk differences. The odds ratio (OR) was obtained via models with random effects (Mantel–Haenszel). Heterogeneity was assessed via the Q test and quantified via the I^2^ value.

**Figure S3. Funnel plot.** Funnel plot of the meta-analysis of the included studies for the primary outcome (anastomotic leak) for the investigation of potential publication bias. Each dot represents the standard error (y-axis) versus the OR of anastomotic leak (x-axis).
